# Supplementary material for: The Dual Prey-Inactivation Strategy of Spiders—In-Depth Venomic Analysis of Cupiennius salei
Source: Toxins (Basel). 2019 Mar 19;11(3):167. doi: 10.3390/toxins11030167 (PMC6468893; doi:10.3390/toxins11030167)
Supplement: Supplementary file 1 [file toxins-11-00167-s001.zip › Supplementary Fig S1.1-S1.19/Supplementary Fig S1.7.pdf]

Supplementary Fig S1.7

CLUSTAL O(1.2.4) multiple sequence alignment: α-Amylases

```
AMY_CUPSA                  ------MAHQI--LFLALGSLVSLTGIACSHHEPQFKSGRTTIVHLFEWKWSDIAEECET
AMY_ALOMA                  -------------------------GVWGSHHEPHFQPGRTTIVHLFEWKWKDIAEECET
AMY_VIRFA                  --------------------------IKCSHHEPNFKPGRTTIVHLFEWKWNDIAEECEI
AMY_ATYPI                  ---------AMLILLLLSAVACQNGFISASNHEPNMKPNRTTTVHLFEWKWNDVAEECEN
tr_A0A1W7RB82_HADSP        MFRYRLKIELAMVIRCLLAFLCVANIVNGSYHEPKTVEGRSVLVHLFEWKWKDIAEECEQ
16816Locus_26668_NEPBR     ------MAGTLISHFALLLFVLSWNCVWSSHHEPHMAGGRTTIVHLFEWKWNDIADECEQ
tr_A0A2P6KNU7_NEPCL        ------MAAGTFSHFAVLLFVLSWNSAWCSHHEPHMAGGRTTIVHLFEWKWNDIADECEQ
tr_A0A087V0Y4_STEMI        ------------------------------------------------------------

AMY_CUPSA                  FLGPYGYGGVQISPPNENGIIWEPFWKTVIHRPWFERYQPVSYKLVTRSGNEDDLRDMVK
AMY_ALOMA                  FLAPKGFGGVQVSPPNENGIVFEPFWNNKIRHPWFERYQPVSYKIATRSGNEDEFRDMVQ
AMY_VIRFA                  FLGPYGYGGVQVSPPNENGIIWEPFWKTVVRRPWFERYQPVSYKLVTRSGNEQEFRDMVR
AMY_ATYPI                  FLGPYGYGAVQVSPANENGVIWMPPWNHDVKRPWFERYQPVSYKIATRSGNELEFREMVR
tr_A0A1W7RB82_HADSP        FLGPYGFGGVQVSPPNENGIVWEPQWDPTIKRPWYERYQPVSYKIATRSGNESEFRDMVR
16816Locus_26668_NEPBR     FLGPYGYGGVQVSPPNENGIVNEPSWNRNVKRPWYERYQPVSYKLITRSGSEMEFRDMVR
tr_A0A2P6KNU7_NEPCL        FLGPYGYGGVQVSPPNENGVVYQPSWNSNVKRPWFERYQPVSYKLITRSGSEMEFRDMVR
tr_A0A087V0Y4_STEMI        ------------------------------------------------------------

AMY_CUPSA                  RCNNAGVRIYVDAVINHMTGHIGAGQGTAGS-SFDPGVPRYDGVPYGPENFNSRDKCPTG
AMY_ALOMA                  RCNNVGVRIYVDSVINHMTGNIGAGQGSGGS-NFDPGALRYEGVPYYAEHFNSQAQCPTG
AMY_VIRFA                  RCNNAGVRIYVDSVINHMTGHIGAGQGTAGS-SFDPGVPRYDGVPYGPEHFNSRDKCPTG
AMY_ATYPI                  RCNNVGVRIYVDSVINHMTGDIGQGHGTGGS-FFDPSNFKYDGVPYSREHFNDKTKCPSK
tr_A0A1W7RB82_HADSP        RCNNAGVRIYADAVINHMTGNIGKGKGTGGS-SFDPEARQYYGVPYGPSDFNSHEECSSE
16816Locus_26668_NEPBR     RCNNAGVRIYVDAVINHMTGNIGAGHGTAGS-YFDPSVPKYDGVPYGPDNFNKGDKCPTG
tr_A0A2P6KNU7_NEPCL        RCNNAGVRIYVDAVINHMTGDIGAGHGTAGS-YFDPSVPKYDGVPYGPDNFNKGDKCPTG
tr_A0A087V0Y4_STEMI        -----------------MTGNIGAGHGTAGS-YFDPVVPKYDGVPYGPEHFNSRDKCPTG
                                            ***.** *:*:.*** ***   :* ****  ..**.  :* :

AMY_CUPSA                  SGDIEDYNNPDQVRNCKLSGLNDLDLGQEYVRNKIADYFNRLIGIGVAGFRVDAAKHMWP
AMY_ALOMA                  SGDIENYNDPQQVRNCKLSGLNDLNLGLEYVRNKIADYMNHLIGIGVAGFRVDAAKHMWP
AMY_VIRFA                  SGDIEDYNNKEQVRNCKLSGLNDLDLGQEYVRNKITAYFNNLISIGVAGFRVDAAKHMWP
AMY_ATYPI                  SGNIEDYNDIFQVRNCQLQGLKDLDLSQGYVRGKIVGYLNKLIDIGVAGFRIDAAKHMWP
tr_A0A1W7RB82_HADSP        SGNIESYQDKYQIRNCMLSGLADLKLSKEYVRGKIVEYLNFLIDIGVAGFRIDASKHMWP
16816Locus_26668_NEPBR     SGDIENYNDKIQVRNCRLSGLADLDLGQEYVRNKIAEYMNKLIDMGVAGFRVDAAKHMWP
tr_A0A2P6KNU7_NEPCL        SGDIENYNDKIQVRNCRLSGLADLDLGQEYVRNKIAGYMNKLIDYGVAGFRVDAAKHMWP
tr_A0A087V0Y4_STEMI        SGDIENYNDKVQVRNCKLSGLNDLDLSQEYVRNKIAEYMNYLIQIGVAGFRVDAAKHMWP
                           **:**.*::  *:*** *.** **.*.  ***.**. *:* **  ******:**:*****

AMY_CUPSA                  GDLSAVYSKMNTLNQTFFP-PGLEPFIYQEVIDLGG-EAIKSQEYVGLGRVTEFRFGKQL
AMY_ALOMA                  HDLQAIYSRLRTLNETVF-PGGKKPFIYQEVIDLGG-EAIKAQEYVGFGRVTEFRYGKHL
AMY_VIRFA                  ADLSAIYSNLNTLNEDFFP-PGTEAFIYQEVIDLGG-EAIKAEEYWSLGRVTEFRYGKHL
AMY_ATYPI                  GDLSAIYGQLKNLNTTFF-DHGKRPYIYQEVIDMGG-EPIKSDQYTSFGRVTEFKYGKKL
tr_A0A1W7RB82_HADSP        GDLKIIYRRLKDLNTEYFS-INTRPFIFQEVIDMGGGEAVRAEEYISLGRVTEFRFGKHL
16816Locus_26668_NEPBR     ADLSAIYGRLHNLKGEVFG-DGKKPLIYQEVIDLGG-EAIKSDEYVAFGRVTEFRYGMNL
tr_A0A2P6KNU7_NEPCL        ADLSAIYGRLHNLKADVFG-DGKKPMIYQEVIDLGG-EAIKSDEYVSFGRVTEFRYGMNL
tr_A0A087V0Y4_STEMI        DDLSIIYNRLNNLNEAYFP-AGRKPFIYQEVIDMGG-EPIKSEEYVEFGRVTEFRFGKYL
                            **. :* .:. *:   *   . .  *:*****:** * :::::*  :******::*  *

AMY_CUPSA                  GDVIRKNYNQLLRYLRNFGEGWSFVSGSNALTFVDNHDNQRGHGAGGYGSILTHKQPRMY
AMY_ALOMA                  GDVIRKNHNQQLRYLRNFGEGWSFVSGSDALTFVDNHDNQRGHGAGGYGAILTFRQPRMY
AMY_VIRFA                  GDVIRKNFNQLLRYLRNFGEEWSFISGSNALTFVDNHDNQRGHGAGGYGSILTHKQSRMY
AMY_ATYPI                  GDVIRKNENQMLKYLKNFGEDWGFTSGNNAVVFLDNHDNQRGHGAGGFGSILTHMEPRMY
tr_A0A1W7RB82_HADSP        GDVIRKNYNQRLKYLKNFGEEWGMVSGHDALTFIDNHDNQRGHGAGGYGSVLTFFDSRMY
16816Locus_26668_NEPBR     GNVIRKNNNQKLKYLRNFGEGWAFQTGNNALAFVDNHDNQRGHGAGGYGSILTHRQSRMY
tr_A0A2P6KNU7_NEPCL        GNVIRKNNNQKLKYLRNFGEGWAFQSGNNALAFVDNHDNQRGHGAGGYGSILTHRQSRMY
tr_A0A087V0Y4_STEMI        GDVIRKNFDQRLKYLRNFGEGWSFVSGSNALTFVDNHDNQRGHGAGGYGSILTHKLPRMY
                           *:***** :* *:**:**** *.: :* :*:.*:*************:*::**.   ***
```

```
AMY_CUPSA                     KMAVAFMLAWPYGLPRVMSSYSWNENIVNGRDENDWIGPPTDSNYNIKNVKRNADLTCGD
AMY_ALOMA                     KIAVAFMLAWPYGLPRVMSSYQWPEYIENGKDINDWVGPPMDDSGNTKDVVRNPDLSCGN
AMY_VIRFA                     KMAVAFMLAWPYGLPRVMSSYSWNENIVNGKDENDWIGPPSDSDYNIKNVTRNPDLTCGD
AMY_ATYPI                     KMGTAFMLAWPYGSPRVMSSYSWPRYIQGGKDLNDWIGPPVDQNYNIKSVTRNADLTCGN
tr_A0A1W7RB82_HADSP           KMATAFMLAWPYGLPRIMSSYKWPRNIESGKDKNDWIGPPHDNEFNIKPVIRHPDFTCGN
16816Locus_26668_NEPBR        KMAVAFMLAWPYGLTRVMSSFSWPENIVNGKDLNDWIGPPHDGNYNIKTVKKNSDLTCGD
tr_A0A2P6KNU7_NEPCL           KMAVAFMLAWPYGLTRVMSSFSWPENIVNGKDLNDWIGPPHDGNYNIKSVKKNSDLTCGD
tr_A0A087V0Y4_STEMI           KMAVAFTLAWPYGLPRVMSSYSWPENIVNGKDTNDWIGPPADSNYNITSVVRNSDLTCGG
                              *:..** ******  *:***:.* . * .*:* ***:*** * . * . * :: *::**.

AMY_CUPSA                     GWVCEHRWRQIYNMVKFRNVAGFEEVHNWWDNGYHQIAFSRGNKGFLAINNENHALDQNL
AMY_ALOMA                     GWICEHRWRQIYNMVKFRNVAGFEEVRDWYDNGFHQIAFGRGNRAFLAINNENFAMNRTL
AMY_VIRFA                     GWVCEHRWRQIYNMVKFRNVACFTTVKNWWDNGYHQIAFARGNKAFIAINNENHSLNQNL
AMY_ATYPI                     GWVCEHRWRQIYNMVKFRNVAGISKMSNWWDNGYQQIAFARQGKGFLAINNENYPLNQRF
tr_A0A1W7RB82_HADSP           GWVCEHRWRQIYSMVKFHNVAGFEPVTFWWDNDYQQIAFGRRGKGFLAINNENYSMDQTL
16816Locus_26668_NEPBR        GWVCEHRWRQIFNMVKFRNVVLFDGVANWWDNGNNQIAFSRGNKGFFAMNNDDHQLNQSL
tr_A0A2P6KNU7_NEPCL           GWVCEHRWRQIFNMVKFRNVVLFDAVANWWDNGNNQIAFSRGNKGFLAMNNDDHQLNQSL
tr_A0A087V0Y4_STEMI           GWVCEHRWRQIYNMVKFRNVVGFETVQNWWDNDYHQIAFSRGSKGFLAINNENHALDQNL
                              **:********:.****:**. :  :  *:**. :****.* .:.*:*:**::. ::: :

AMY_CUPSA                     NTGLPEGTYCDVISGNKDGNQCTGRSVAVSSSGHAQIFVDNAWEDPMIAIHVEAKL-
AMY_ALOMA                     HTELPEGTYCDVISGNKEGDACTGRTVQVSSTGHAQIYIDNNWEDPIIAIHVEAKL-
AMY_VIRFA                     ETGLPEGTYCDVISGNKDGNRCTGRSVQVDNSGYAQIVVENSWEDPMIAIHVEAKL-
AMY_ATYPI                     QTTLPQGKYCDVISGNKEGGRCTGRAVDVDASGEAQLYVSNTWDDPMVALHLEAKL-
tr_A0A1W7RB82_HADSP           PTGLPPGTYCDVISGKVEDNKCTGRSVNVGHDGKAKIFVDSTWEDPMLAIHVEAKLN
16816Locus_26668_NEPBR        QTGLPEGTYCDVISGNKDGNRCTGRSVQVGRDGRAQIHIDNSAGDPMIALHIEAKL-
tr_A0A2P6KNU7_NEPCL           QTGLPEGTYCDVISGNKEGNRCTGRSVQVGSDGKARIHIDTSAEDPMIALHVQAKL-
tr_A0A087V0Y4_STEMI           QTGLPEGTYCDVISGNRDGGQ------------------------------------
                               * ** *.*******: :..
```

Percent Identity Matrix - created by Clustal2.1

1: AMY_CUPSA	100.00	78.50	87.97	69.10	67.49	75.24	75.43	84.21
2: AMY_ALOMA	78.50	100.00	77.27	67.32	69.03	72.39	72.39	75.56
3: AMY_VIRFA	87.97	77.27	100.00	72.73	70.22	77.91	78.11	81.99
4: AMY_ATYPI	69.10	67.32	72.73	100.00	66.54	68.45	69.41	72.50
5: tr_A0A1W7RB82_HADSP	67.49	69.03	70.22	66.54	100.00	65.65	65.28	69.81
6: 16816Locus_26668_NEPBR	75.24	72.39	77.91	68.45	65.65	100.00	93.93	79.78
7: tr_A0A2P6KNU7_NEPCL	75.43	72.39	78.11	69.41	65.28	93.93	100.00	79.78
8: tr_A0A087V0Y4_STEMI	84.21	75.56	81.99	72.50	69.81	79.78	79.78	100.00

Proteomic coverage trypsin

>AMY_CUPSA alpha amylase

```
MAHQILFLAL GSLVSLTGIA CSHHEPQFKS GRTTIVHLFE WKWSDIAEEC ETFLGPYGYG GVQISPPNEN GIIWEPFWKT
VIHRPWFERY QPVSYKLVTR SGNEDDLRDM VKRCNNAGVR IYVDAVINHM TGHIGAGQGT AGSSFDPGVP RYDGVPYGPE
NFNSRDKCPT GSGDIEDYNN PDQVRNCKLS GLNDLDLGQE YVRNKIADYF NRLIGIGVAG FRVDAAKHMW
PGDLSAVYSK MNTLNQTFFP PGLEPFIYQE VIDLGGEAIK SQEYVGLGRV TEFRFGKQLG DVIRKNYNQL LRYLRNFGEG
WSFVSGSNAL TFVDNHDNQR GHGAGGYGSI LTHKQPRMYK MAVAFMLAWP YGLPRVMSSY SWNENIVNGR DENDWIGPPT
DSNYNIKNVK RNADLTCGDG WVCEHRWRQI YNMVKFRNVA GFEEVHNWWD NGYHQIAFSR GNKGFLAINN ENHALDQNLN
TGLPEGTYCD VISGNKDGNQ CTGRSVAVSS SGHAQIFVDN AWEDPMIAIH VEAKL
```

Proteomic coverage chymotrypsin

>AMY_CUPSA alpha amylase

```
MAHQILFLAL GSLVSLTGIA CSHHEPQFKS GRTTIVHLFE WKWSDIAEEC ETFLGPYGYG GVQISPPNEN GIIWEPFWKT
VIHRPWFERY QPVSYKLVTR SGNEDDLRDM VKRCNNAGVR IYVDAVINHM TGHIGAGQGT AGSSFDPGVP RYDGVPYGPE
```

NFNSRDKCPT GSGDIEDYNN PDQVRNCKLSGLNDLDLGQE YVRNKIADYF NRLIGIGVAG FRVDAAKHMW PGDLSAVYSK
MNTLNQTFFP PGLEPFIYQE VIDLGGEAIK SQEYVGLGRV TEFRFGKQLG DVIRKNYNQL LRYLRNFGEG WSFVSGSNAL
TFVDNHDNQR GHGAGGYGSI LTHKQPRMYK MAVAFMLAWP YGLPRVMSSY SWNENIVNGR DENDWIGPPT DSNYNIKNVK
RNADLTCGDG WVCEHRWRQI YNMVKFRNVA GFEEVHNWWD NGYHQIAFSR GNKGFLAINN ENHALDQNLN TGLPEGTYCD
VISGNKDGNQ CTGRSVAVSS SGHAQIFVDN AWEDPMIAIH VEAKL

Fragments of AMY_CUPSA

Proteomic coverage trypsin

>AMY_fragment_1_CUPSA alpha amylase fragment 1 [MH795778]

MAHQILFLAL GSLVSLTGIA CSHHEPQFKS GRTTIVHLFE WKWSDIAEEC ETFLGPYGYG GVQISPPNEN GIIWEPFWKT
VIHRPWFERY QPVSYKLVTR SGNEDDLRDM VKRCNNAGVR IYVDAVINHM TGHIGAGQGT AGSSFDPGVP RYDGVPYGPE
NFNSRDKCPT GSGDIEDYNN PDQVRNCKLS GLNDLDLGQE YVRNKIADYF NRLIGI

Proteomic coverage chymotrypsin

>AMY_fragment_1_CUPSA alpha amylase fragment 1 [MH795778]

MAHQILFLAL GSLVSLTGIA CSHHEPQFKS GRTTIVHLFE WKWSDIAEEC ETFLGPYGYG GVQISPPNEN GIIWEPFWKT
VIHRPWFERY QPVSYKLVTR SGNEDDLRDM VKRCNNAGVR IYVDAVINHM TGHIGAGQGT AGSSFDPGVP RYDGVPYGPE
NFNSRDKCPT GSGDIEDYNN PDQVRNCKLSGLNDLDLGQE YVRNKIADYF NRLIGI

Proteomic coverage trypsin

>AMY_fragment_2_S1_CUPSA alpha amylase fragment 2 [MH795779/MH795780]

MAHQILFLAL VSLVSLTGIA CSHHEPQFKS GRTTIVHLFE WKWSDIAEEC ETFLGPYGYG GVQISPPNEN GIIWEPFWKT
VIHRPWFERY QPVSYKLVTR SGNEDDLRDM VKRCNNAGVR IYVDAVINHM TGHIGAGQGT AGSSFDPGVP RYDGVPYGPE
NFNSRDKCPT GSGDIEDYNN PDQVRNCKLS GLNDLDLGQE YVRNKIADYF NRLIGI

Proteomic coverage chymotrypsin

>AMY_fragment_2_S1_CUPSA alpha amylase fragment 2 [MH795779/MH795780]

MAHQILFLAL VSLVSLTGIA CSHHEPQFKS GRTTIVHLFE WKWSDIAEEC ETFLGPYGYG GVQISPPNEN GIIWEPFWKT
VIHRPWFERY QPVSYKLVTR SGNEDDLRDM VKRCNNAGVR IYVDAVINHM TGHIGAGQGT AGSSFDPGVP RYDGVPYGPE
NFNSRDKCPT GSGDIEDYNN PDQVRNCKLSGLNDLDLGQE YVRNKIADYF NRLIGI

Proteomic coverage trypsin

>AMY_fragment_3_CUPSA alpha amylase fragment 3 [MH795781]

MAHQILFLAL GCLVSLTGIA CSHHEPQFKS GRTTIVHLFE WKWSDIAEEC ETFLGPYGYG GVQISPPNEN GIIWEPFWKT
VIHRPWFERY QPVSYKLVTR SGNEDDLRDM VKRCNNAGVR IYVDAVINHM TGHIGAGQGT AGSSFDPGVP RYD

Proteomic coverage chymotrypsin

>AMY_fragment_3_CUPSA alpha amylase fragment 3 [MH795781]

MAHQILFLAL GCLVSLTGIA CSHHEPQFKS GRTTIVHLFE WKWSDIAEEC ETFLGPYGYG GVQISPPNEN GIIWEPFWKT
VIHRPWFERY QPVSYKLVTR SGNEDDLRDM VKRCNNAGVR IYVDAVINHM TGHIGAGQGT AGSSFDPGVP RYD

Proteomic coverage trypsin

>AMY_fragment_4_CUPSA alpha amylase fragment 4 [MH795782]

MAHQILFLAL GSLVSLTGIA CSHHEPQFKS GR**TTIVHLFE WKWSDIAEEC ETFLGPYGYG GVQ**ISSPN**EN GIIWEPFWKT VIHRPWFERY QPVSYKLVTR** SG

Proteomic coverage chymotrypsin

>AMY_fragment_4_CUPSA alpha amylase fragment 4 [MH795782]

MAHQILFLAL GSLVSLTGIA C**SHHEPQFKS GRTTIVHLFE WKWSDIAEEC ETF**LGPYGYG GVQISSPNEN GIIW**EPFWKT VIHRPWFERY QPVSY**KLVTR SG

Proteomic coverage trypsin

>AMY_fragment_5_CUPSA alpha amylase fragment 5 [MH795783]

D**IEDYNNPDQ VRNCKLSGLN DLDLGQEYVR NKIADYFNRL IGIGVAGFRV DAAKHMWPGD LSAVYSK**MNT LNQSFFPPGL EPF**IYQEVID LGGE**

Proteomic coverage chymotrypsin

>AMY_fragment_5_CUPSA alpha amylase fragment 5 [MH795783]

DIE**DYNNPDQ VRNCKL**SGL**N DLDLGQEYVR NKIADYFNRL IGIGVAGFRV DAAKHMWPGD LSAVY**SKMNT LNQSFFPPGL EPFIYQEVID LGGE

Proteomic coverage trypsin

>AMY_fragment_6a_CUPSA alpha amylase fragment 6a [MH795784]

D**IEDYNNPDQ VRNCKLSGLN DLDLGQEYVR** I**KIADYFNRL IGIGVAGFRV DAAKHMWPGD LSAVYSK**MNT LNQTFFPPGL EPF**IYQEVID LGGE**

Proteomic coverage chymotrypsin

>AMY_fragment_6a_CUPSA alpha amylase fragment 6a [MH795784]

DIE**DYNNPDQ VRNCKL**SGL**N DLDLGQEY**VR IKIADY**FNRL IGIGVAGFRV DAAKHMWPGD LSAVY**SKMNT LNQTFFPPGL EPFIYQEVID LGGE

Proteomic coverage trypsin

>AMY_fragment_6b_CUPSA alpha amylase fragment 6b [MH795785]

D**IEDYNNPDQ VRNCKLSGLN DLDLGQEYVR** I**KIADYFNRL IGIGVAGFRV DAAKHMWPGD LSAVYSK**MNT LNQSFFPPGL EPF**IYQEVID LGGE**

Proteomic coverage chymotrypsin

>AMY_fragment_6b_CUPSA alpha amylase fragment 6b [MH795785]

DIE**DYNNPDQ VRNCKL**SGL**N DLDLGQEY**VR IKIADY**FNRL IGIGVAGFRV DAAKHMWPGD LSAVY**SKMNT LNQSFFPPGL EPFIYQEVID LGGE

Proteomic coverage trypsin

>AMY_fragment_7a_CUPSA alpha amylase fragment 7a [MH795786]

DIEDYNNPDQ VRNCKLSGLN DLDLGQEYVR IKLADYFNRL IGIGVAGFRV DAAKHMWPGD LSAVYSKMNT LNQTFFPPGL EPFIYQEVID LGGE

Proteomic coverage chymotrypsin

>AMY_fragment_7a_CUPSA alpha amylase fragment 7a [MH795786]

DIEDYNNPDQ VRNCKLSGLN DLDLGQEYVR IKLADYFNRL IGIGVAGFRV DAAKHMWPGD LSAVYSKMNT LNQTFFPPGL EPFIYQEVID LGGE

Proteomic coverage trypsin

>AMY_fragment_7b_CUPSA alpha amylase fragment 7b [MH795787]

DIEDYNNPDQ VRNCKLSGLN DLDLGQEYVR IKLADYFNRL IGIGVAGFRV DAAKHMWPGD LSAVYSKMNT LNQSFFPPGL EPFIYQEVID LGGE

Proteomic coverage chymotrypsin

>AMY_fragment_7b_CUPSA alpha amylase fragment 7b [MH795787]

DIEDYNNPDQ VRNCKLSGLN DLDLGQEYVR IKLADYFNRL IGIGVAGFRV DAAKHMWPGD LSAVYSKMNT LNQSFFPPGL EPFIYQEVID LGGE

Proteomic coverage trypsin

>AMY_fragment_8_CUPSA alpha amylase fragment 8 [MH795788]

YGSILTHKQP RMYKMAVAFM LAWPYGLPRV MSSYSWNENI VNGRDENDWI GPPTDSNYNI KNVKKNADLT CGDGWVCEHR WRQIYNMVKF RNVAGFEEVH NWWDNGYHQI AFSRGNKGFL AINNENHALD Q

Proteomic coverage chymotrypsin

>AMY_fragment_8_CUPSA alpha amylase fragment 8 [MH795788]

YGSILTHKQP RMYKMAVAFM LAWPYGLPRV MSSYSWNENI VNGRDENDWI GPPTDSNYNI KNVKKNADLT CGDGWVCEHR WRQIYNMVKF RNVAGFEEVH NWWDNGYHQI AFSRGNKGFL AINNENHALD Q

Proteomic coverage trypsin

>AMY_fragment_9_CUPSA alpha amylase fragment 9 [MH795789]

NGYHQIAFSR GNKGFLAINN ENHALDQNLN TGLPEGTYCD VISGNKDGNQ CTGRSVAVSS SGHAQIFVDN VWEDPMIAIH VEAKL

Proteomic coverage chymotrypsin

>AMY_fragment_9_CUPSA alpha amylase fragment 9 [MH795789]

NGYHQIAFSR GNKGFLAINN ENHALDQNLN TGLPEGTYCD VISGNKDGNQ CTGRSVAVSS SGHAQIFVDN VWEDPMIAIH VEAKL

Supplementary Fig S1.7 Multiple sequence alignment of α-amylase from the venom glands of *C. salei* (AMY_CUPSA [MH795777]), *A. marikovskyi* (AMY_ALOMA [xxxx]), *V. fasciatus* (AMY_VIRFA [xxxx]), *Atypus piceus* (AMY_ATYPI [xxxx]), the scorpion *Hadrurus spadix* (tr_A0A1W7RB82_HADSP), from the midgut transcriptome of *Nephilingis cruentata* (16816Locus_26668_NEPBR, [58]), from genomic DNA of *Nephila clavipes* (tr_A0A2P6KNU7_NEPCL), and from *S.*

mimosarum (tr_A0A087V0Y4_STEMI). Signal peptides are marked in black characters, cysteines in white with a black box, positively charged amino acids in pink, negatively charged amino acids in blue, hydrophobic amino acids in red and polar amino acids in green. Ca^{2+} binding sites are highlighted in gray and catalytic residues in yellow.
